# ACCELERATED CHILDHOOD SKELETAL AGING IS PROTECTIVE OF DEVELOPMENT OF SARCOPENIA IN LATER LIFE

**DOI:** 10.1093/geroni/igz038.2267

**Published:** 2019-11-08

**Authors:** Matthew J Peterson, Andrew W Froehle

**Affiliations:** 1 Campbell University, Buies Creek, North Carolina, United States; 2 Wright State University, Dayton, Ohio, United States

## Abstract

Sarcopenia is an age-related loss of muscle mass and strength that has a multitude of adverse sequelae. Similar to other aging-related phenomenon, sarcopenia is likely the product of inputs that begin in utero and continue throughout the lifespan. We hypothesized that patterns of childhood skeletal growth predict sarcopenia status later in life. Data are from N=202 lifelong participants of the Fels Longitudinal Study (median lifetime visits=33). At the sarcopenia measure visit, participants were aged 65.8 + 10.3 years, 54% female, with body mass index of 27.5 + 4.9. Sarcopenia was defined using published sex-specific cutpoints from dual energy x-ray absorptiometry quantified appendicular lean mass/height2. Childhood skeletal age was calculated from serial hand-wrist radiographs (FELS method). Residual skeletal aging (RSA) was calculated as skeletal age minus predicted chronological age at peak height growth velocity during adolescence. RSA variance was similar in both sexes, with a range of -2 (delayed skeletal aging) to +2 years (accelerated skeletal aging). In older age, 6% of males and 22% of females exhibited sarcopenia. In multivariate logistic regression models controlling for age, self-reported physical activity, and grip strength (all measured at sarcopenia visit), accelerated RSA was protective of sarcopenia (Adjusted OR=0.58; 95% CI: 0.35-0.94). This is the first study to link childhood skeletal maturation to sarcopenia later in life. Biological pathways that explain this association likely include physiological, environmental, and genetic factors that facilitate communication between bone and muscle, and span the life course. Determining their influence is the next important step in this work.

